# Implementation of Bilateral Rectus Sheath Blocks in Conjunction With Transversus Thoracis Plane and Pectointercostal Fascial Blocks for Immediate Postoperative Analgesia After Cardiac Surgery

**DOI:** 10.7759/cureus.26592

**Published:** 2022-07-05

**Authors:** Lauren Everett, TomMario A Davis, Seema P Deshpande, Samhati Mondal

**Affiliations:** 1 Department of Anesthesiology, University of Maryland School of Medicine, Baltimore, USA

**Keywords:** multimodality pain management, ultrasound-guided regional anesthesia, rectus sheath block, chest tube, postoperative pain relief, adult cardiac surgery

## Abstract

Pain continues to be a well-known complication of cardiac surgery in the postoperative period and intravenous opioid analgesia has traditionally been employed to manage cardiac surgical pain. However, both components have contributed to a multitude of undesirable adverse effects which can further exacerbate delays in recovery. Often overlooked in the analgesic plan, chest tube pain contributes significantly to the overall postoperative pain from cardiac surgery. Novel regional anesthetic blocks have shown great promise as analgesic adjuncts for cardiothoracic anesthesia but preliminary investigations focus primarily on management of sternotomy pain. Reduction of chest tube pain should be considered while implementing regional blocks to control surgical pain. This study presents a case where the rectus sheath block minimized chest tube pain after aortic valve replacement in conjunction with intercostal nerve blocks and a multimodal analgesic plan.

## Introduction

Inadequately controlled pain after cardiac surgery is the leading cause of postoperative complications and poses a significant barrier to timely recovery [1]. Acute postoperative pain that is inadequately managed leads to a heightened sympathetic response, prolonged immobilization, interference with respiratory physiology, delirium, and increased risk of developing chronic postoperative pain [1,2]. Poststernotomy pain commonly makes up the majority of complaints but additionally patients suffer arm, back, and chest tube pain [1-3]. A study comparing acute postoperative pain severity and location in patients with shortened versus prolonged time-to-removal of chest tubes determined that epigastric pain was the most commonly reported site, after the sternum, indicating the impact of subxiphoid chest tubes on pain experiences [4]. Pain scores remain severe in the postoperative period, especially exacerbated by coughing, mobilization, and deep breathing [2]. The impact of chest tube pain on postoperative pulmonary toileting is significant and places patients at risk for pulmonary complications, such as pneumonia, atelectasis, pleural effusions, and hypoxemia [5]. Early removal of chest tubes has been shown to decrease pain severity in the epigastrium compared to patients with prolonged drainage further emphasizing the need for adequate chest tube analgesia while the drains remain in place [4].

Traditionally, intravenous (IV) opioid analgesia has been employed to manage cardiac surgical pain both intra- and postoperatively but at the expense of a multitude of undesirable adverse effects which can further exacerbate delays in recovery [1]. Investigation into multimodal analgesic management has expanded recently with the intention of discovering new efficacious methods to treat postoperative cardiac surgical pain. The introduction of novel regional anesthetic blocks, including transversus thoracis muscle plane (TTP), pectointercostal fascial (PIF), and erector spinae plane (ESP) blocks, has shown great promise in preliminary studies as analgesic adjuncts for cardiothoracic anesthesia but more randomized clinical trials will be required to solidify the inference.

Despite showing promising results for relief of sternotomy pain with ESP, TTP, and PIF blocks, subxiphoid or epigastric pain continues to be a problem for postcardiac surgical patients. Concerning subxiphoid pain related to epigastric chest tubes, Sepolvere et al. observed positive results regarding pain and pulmonary capacity in a case series following cardiac surgery patients postoperatively who received rectus sheath (RS) blocks [3]. Yamamoto et al. describe encouraging observational results when combining TTP and RS blocks preoperatively in the pediatric cardiac surgery population [6]. However, these novel analgesic blocks have not been studied extensively in conjunction with each other in adults undergoing cardiac surgery.

We report a case where bilateral RS blocks minimized chest tube-induced pain after aortic valve replacement in conjunction with intercostal nerve blocks and a multimodal analgesic plan. Written Health Insurance Portability and Accountability Act (HIPAA) authorization and informed consent have been obtained from the patient.

## Case presentation

A 31-year-old young male (46 kg, 152 cm) underwent aortic valve replacement by sternotomy for aortic stenosis secondary to a bicuspid valve. Being young and otherwise healthy, he was a perfect candidate to achieve enhanced recovery after cardiac surgery (ERACS). To manage acute postoperative pain commonly experienced in cardiac surgery patients, we employed three blocks - bilateral TTP and PIF blocks for sternal irritation and bilateral RS blocks to combat epigastric chest tube pain.

**Figure 1:**
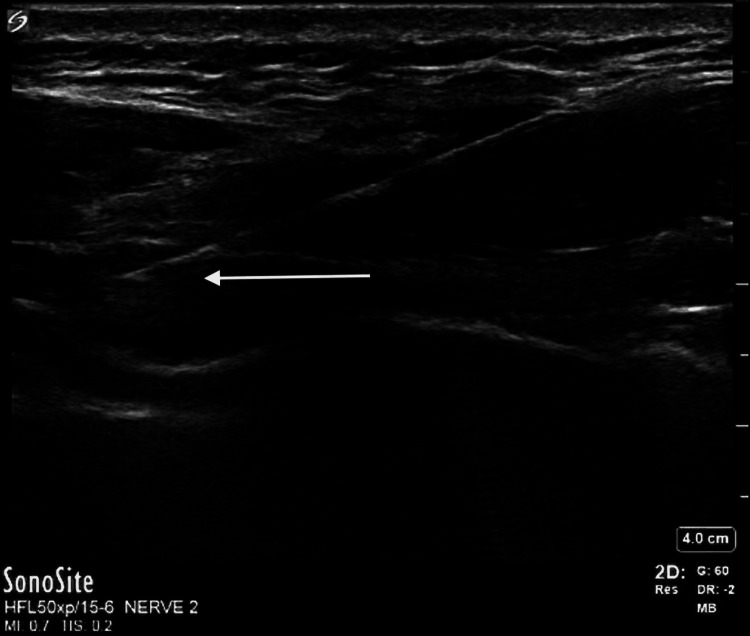
Rectus sheath block Arrow pointing to needle tip injecting local anesthetic into posterior sheath of rectus abdominus between the rectus abdominus muscle (superficial) and peritoneal cavity (deep).

**Figure 2:**
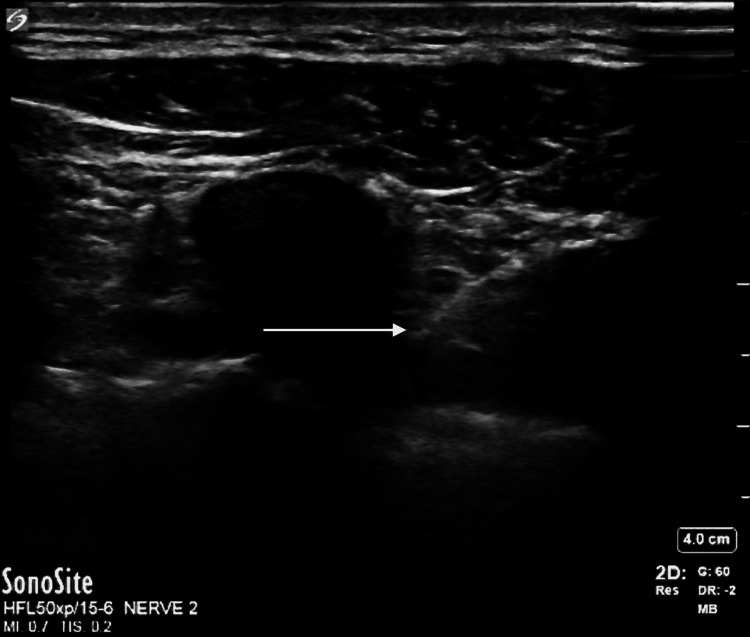
Transversus thoracis muscle plane block Arrow pointing to needle tip injecting local anesthetic at the fourth and fifth intercostal spaces into the fascial plane between the internal intercostal (superficial) and transversus thoracis (deep) muscles.

**Figure 3:**
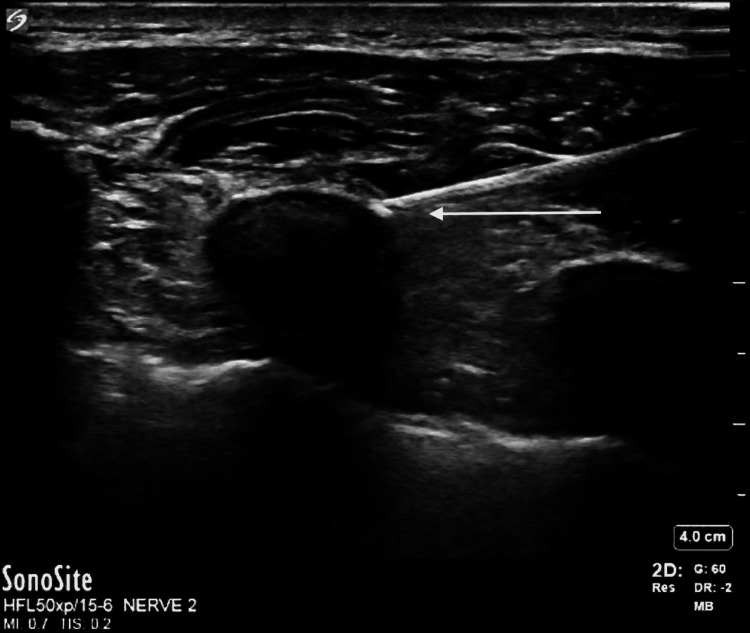
Pectointercostal fascial block Arrow pointing to needle tip injecting local anesthetic between the pectoralis major (superficial) and intercostal (deep) muscles.

Preoperatively, the patient received 1 g of oral acetaminophen. IV ketamine and dexmedetomidine infusions, at 5 mcg/kg/min and 0.2 mcg/kg/h, respectively, were started 15 minutes before incision, and continued throughout the case. A total of 600 mcg fentanyl was administered up until the blocks. Requiring 10 min for execution, bilateral TTP (15 cc) and PIF (15 cc) blocks were performed followed by bilateral RS blocks (10 cc total, 5 cc on each side) under ultrasound guidance at the end of the case (Figures 1-3). Using a 21-gauge, 50 mm needle, a total of 40 ml of 0.25% bupivacaine with epinephrine was injected in incremental single shot amounts after confirmation of negative aspiration.

The patient was transferred to the intensive care unit (ICU). All sedation was paused after verbal handoff with the ICU team. The patient passed a spontaneous breathing trial (SBT) 3.5 hours after admission to the ICU and was successfully extubated after four hours. The patient appeared visibly alert, oriented, yet comfortable, and calm before and after extubation. The patient tolerated incisional dressing changes well and reported more discomfort near the upper sternal incision compared to the chest tube sites. On postoperative day (POD) 1, the patient was tolerating ambulation, positional changes, physical therapy, and deep breathing exercises, and was transferred to a stepdown unit. Numerical pain scores (0-10) reported by nursing staff over the first 24 hours ranged primarily from 4 to 6 with a few reports of 0 and 8. He required only 0.5 mg of hydromorphone in the first 11 hours postoperatively, after which his pain score appeared to increase, and he received an additional 1.5 mg of hydromorphone within the first 24 hours since the time of block placement. Afterward, he was transitioned to 5 mg of oral oxycodone which gradually decreased until POD 4. Per discussion with the patient on POD 1 and 4, the sternotomy pain was most noticeable at the upper sternum, but overall incisional pain was tolerable and well managed. The block effects appeared to wane after 12 hours as there was a sharp increase in opioid requirement although the patient reported increased pain not until after 24 hours. A temporary increase in reported numerical pain scores (7-9) was also observed after 24 hours. Chest tubes and pacer wires were removed on POD 4 without any discomfort. No acute or chronic complications occurred secondary to the blocks.

## Discussion

Our case highlights a promising regional analgesic approach to sternotomy within a larger multimodal analgesic plan since severe pain in the postoperative period can be exacerbated by coughing, mobilization, and deep breathing and places patients at risk for pulmonary complications, such as pneumonia, atelectasis, pleural effusions, and hypoxemia [2,5]. The majority of single block studies available include a multimodal pain regimen in addition to the regional anesthetic indicating the importance of blocks as an adjunct in a larger pain management plan. The three blocks reported in our case required limited time, equipment, and no repositioning for successful application. These blocks are relatively safe and in a study of 299 patients receiving TTP blocks, only two patients developed superficial infections [1]. Regardless, potential complications include local anesthetic toxicity, injection into vasculature, pneumothorax, and anaphylaxis [1].

It should be noted that our case observed a relatively healthy young adult. Patients younger than 60 years old reportedly have more severe acute postoperative pain [2]. However, many patients presenting for cardiac surgery often are elderly and have multiple comorbidities, including chronic pain and pulmonary comorbidities which might pose challenges to the efficacy of the blocks. Cibelli et al. employed the catheter technique described by Chanowski et al. but as a pectoralis intercostal RS continuous blockade in a patient with fibromyalgia and history of chronic opioid use [7,8]. The case reported a limited need for postoperative opioids and the patient was discharged home with oral gabapentin [7]. Tailoring the block type and location to the patient, surgical plan, and chest tube exit sites may impact postoperative pain outcomes. For example, myofascial syndrome drastically increases in patients who undergo internal mammary artery dissection increasing the rate of chronic postoperative pain and necessitating a more aggressive pain management plan [2]. In addition, the TTP block may have limited use in prior sternotomy patients due to poor anesthetic spread secondary to tissue plane remodeling [1].

We were limited in the maximum allowable local anesthetics (LA) dose and volume due to the low body weight of our patient. As pain caused by chest tubes continues to be debilitating and impairs postcardiac surgical deep breathing exercises, we chose to use liberal volume for the RS block (5 cc on each side) which left us with only 15 cc of LA for both TTP and PIF blocks bilaterally [2,4,5]. As a result, we could not cover upper sternal areas with PIF blocks, which have shown to spread and work better when injected at multiple sites (upper, middle, and lower sternal areas) [9]. Although the American Society of Regional Anesthesia (ASRA) reported that TTP blocks are primarily volume blocks, it is possible to still use only a small volume of LA to produce efficacious analgesia [10]. Additionally, in our case, visualization of the myofascial plane was obscured postoperatively and a satisfactory spread of LA was not visualized. Our patient reportedly pointed to more pain in the upper sternal region compared to mid to low sternal areas and surprisingly little to no pain at the chest tube sites. Overall, our case shows the RS block can effectively reduce chest tube-induced pain in postoperative cardiac patients and enable them to engage in rehabilitation and participate in deep breathing exercises more efficiently. Further research investigating this pain management approach in cardiac surgery patients is essential to validate the analgesic efficacy of this regional technique. Ultimately, this has the potential to help achieve enhanced recovery as our patient did. The ICU length of stay was shortened to less than 24 hours and without any postoperative pulmonary complications. The patient did not experience any opioid-induced side effects including postoperative nausea, vomiting, or gastroparesis.

## Conclusions

RS blocks in adult postcardiac surgical patients can attenuate chest tube pain and enhance recovery. This, in combination with other regional blocks and a multimodal medication plan, has exhibited great potential to become routine analgesic adjuncts for cardiothoracic anesthesia. The blocks are fast, safe, and easily accessible. Controlled, randomized studies in larger patient populations are needed to further investigate the potential benefits.
